# Regulation of stem cell identity by miR-200a during spinal cord regeneration

**DOI:** 10.1242/dev.200033

**Published:** 2022-02-14

**Authors:** Sarah E. Walker, Keith Z. Sabin, Micah D. Gearhart, Kenta Yamamoto, Karen Echeverri

**Affiliations:** 1 Eugene Bell Center for Regenerative Biology and Tissue Engineering, Marine Biological Laboratory, Woods Hole, MA 02543, USA; 2 University of Minnesota, Minneapolis, MN 55455, USA

**Keywords:** Axolotl, Spinal cord, Stem cell, Mesoderm, Regeneration

## Abstract

Axolotls are an important model organism for multiple types of regeneration, including functional spinal cord regeneration. Remarkably, axolotls can repair their spinal cord after a small lesion injury and can also regenerate their entire tail following amputation. Several classical signaling pathways that are used during development are reactivated during regeneration, but how this is regulated remains a mystery. We have previously identified miR-200a as a key factor that promotes successful spinal cord regeneration. Here, using RNA-seq analysis, we discovered that the inhibition of miR-200a results in an upregulation of the classical mesodermal marker *brachyury* in spinal cord cells after injury. However, these cells still express the neural stem cell marker *sox2*. *In vivo* cell tracking allowed us to determine that these cells can give rise to cells of both the neural and mesoderm lineage. Additionally, we found that miR-200a can directly regulate *brachyury* via a seed sequence in the 3′UTR of the gene. Our data indicate that miR-200a represses mesodermal cell fate after a small lesion injury in the spinal cord when only glial cells and neurons need to be replaced.

## Abstract (precis)

**Summary:** Axolotl spinal cord cells have the potential to form cells of the ectoderm and mesoderm depending on the extent of the injury they are responding to.

## INTRODUCTION

Regeneration has been observed throughout the plant and animal kingdoms for many years (Sanchez Alvarado and Tsonis, 2006). Among vertebrates, the Mexican axolotl salamander has the remarkable ability to faithfully regenerate its spinal cord after injury. This process has been most commonly studied in the context of tail amputation (McHedlishvili et al., 2012; Monaghan et al., 2007; Piatt, 1955; Rodrigo Albors et al., 2015; Zhang et al., 2000, 2002), but the axolotl spinal cord also regenerates after a more-targeted transection injury (Butler and Ward, 1965, 1967; Clarke et al., 1988; O'Hara et al., 1992; Zukor et al., 2011). These lines of investigation have identified a population of Sox2^+^/GFAP^+^ glial cells that function as bona fide neural stem cells (NSCs) in the axolotl spinal cord (Echeverri and Tanaka, 2002; Fei et al., 2014; 2016; McHedlishvili et al., 2012; Rodrigo Albors et al., 2015). These NSCs are crucial for regenerative repair, as they proliferate after injury and differentiate into new glia and neurons (Echeverri and Tanaka, 2002; McHedlishvili et al., 2012; Rodrigo Albors et al., 2015). Inhibition of NSC function by CRISPR/Cas9-mediated knockout of Sox2 results in deficient regenerative outgrowth of the spinal cord after tail amputation (Fei et al., 2014, 2016), indicating the importance of NSCs to successful spinal cord regeneration.

Early experiments aimed at determining the potential of GFAP^+^/Sox2^+^ NSCs prospectively labeled these cells with the glial fibrillary acidic protein (GFAP) promoter driving GFP expression and used *in vivo* fluorescence imaging to follow GFP^+^ glial cells after tail amputation. Most GFP^+^ NSCs gave rise to new neurons and glia, but a small proportion of labeled cells left the spinal cord and contributed to muscle and cartilage within the regenerated tail (Echeverri and Tanaka, 2002). Similar experiments using grafting of GFP^+^ spinal cords into non-transgenic animals further confirmed that spinal cord cells exited the spinal cord and contributed to cells of other lineages during tail regeneration (McHedlishvili et al., 2007).

Recent reports have identified a population of progenitors, called neuromesodermal progenitors (NMPs), that reside in the posterior of developing vertebrate embryos (Henrique et al., 2015; Kimelman, 2016b). NMPs are competent to contribute to both the mesoderm and spinal cord during embryonic development (Garriock et al., 2015; Henrique et al., 2015; Tzouanacou et al., 2009); however, their potential role in regeneration has not yet been determined. Extensive genetic and biochemical analysis determined that NMPs can be defined by the co-expression of low levels of the transcription factors brachyury and Sox2 (Gouti et al., 2017; Koch et al., 2017; Turner et al., 2014; Wymeersch et al., 2016). Moreover, the relative levels of Fgf and Wnt signaling activity regulate NMP cell fate decisions (i.e. differentiation into mesodermal progenitors or neural progenitors) (Bouldin et al., 2015; Garriock et al., 2015; Goto et al., 2017; Gouti et al., 2015, 2017; Martin, 2016; Martin and Kimelman, 2008; Turner et al., 2014). Interestingly, both Fgf and Wnt signaling are also important regulators of the NSC response to tail amputation, as inhibition of either Wnt or Fgf blocks tail regeneration (Makanae et al., 2016; Ponomareva et al., 2015; Zhang et al., 2000; Albors et al., 2015). The role of individual Fgf ligands in spinal cord regeneration is relatively unknown, whereas the expression of Wnt5 has been elegantly shown to be essential for oriented cell division and outgrowth of the spinal cord after tail amputation. However, the activity of these pathways after spinal cord transection has not been well characterized. Collectively, these findings indicate that both NMP and NSCs can give rise to cells of an ectodermal and mesodermal lineage, and may potentially use similar signaling pathways to determine cell fate decisions. However, no work to date has identified a role for NMPs in a regenerative context, nor have the underlying molecular signals that may regulate both NSC and NMP cell fate decisions been identified.

To date, a few molecular signals that are required for NSC responses to injury have been identified after both tail amputation and spinal cord transection. Sonic hedgehog, Wnt/PCP and Fgf signaling are indispensable for the pro-regenerative NSC response to tail amputation (Rodrigo Albors et al., 2015; Schnapp et al., 2005; Zhang et al., 2000, 2002). During spinal cord regeneration after transection, the transcriptional complex AP-1^cFos/JunB^ and MAP kinase signaling are crucial regulators of the NSC response to injury (Sabin et al., 2015, 2019). Additionally, microRNA (miRNA) signaling is important to fine-tune the NSC response to injury after both tail amputation and spinal cord transection (Diaz Quiroz et al., 2014; Gearhart et al., 2015; Lepp and Carlone, 2014; Sehm et al., 2009).

Recent work (Sabin et al., 2019) has uncovered an important role for miR-200a in regulating NSC responses to a transection injury in the axolotl spinal cord. After injury, miR-200a is upregulated in NSCs, where it directly represses *c-jun* expression to promote a pro-regenerative glial cell response. Moreover, miR-200a inhibition using an antisense inhibitor led to an increase in expression of genes implicated in glial scar formation and resulted in defects in axonal regrowth, indicating the importance of this miRNA in spinal cord regeneration.

Although previous work uncovered a novel role for miR-200a in regulating spinal cord regeneration, the function of miR-200a has been most extensively studied during neurodevelopment and epithelial-to-mesenchymal transition (EMT) (Trumbach and Prakash, 2015; Zaravinos, 2015). miR-200a inhibits EMT by directly repressing the expression of the transcription factor *β-catenin* (Su et al., 2012; Zaravinos, 2015), leading to maintained epithelial polarity and decreased Wnt signaling. During neurodevelopment, miR-200 family members regulate many processes, including: neuronal survival (Karres et al., 2007), neuroepithelial progenitor proliferation, NSC identity and neuroblast transition (Morante et al., 2013), and neural progenitor identity and cell cycle dynamics (Peng et al., 2012). The miR-200 family also fine-tunes signaling networks necessary for neurogenesis (Choi et al., 2008; Vallejo et al., 2011) and gliogenesis (Buller et al., 2012). These studies have provided extensive evidence that miR-200a regulates various developmental processes involved in determining cell fate. Whether miR-200a regulates such processes in NSCs during axolotl spinal cord regeneration remains unknown.

In this study, we identify a role for miR-200a in stabilizing the NSC identity after spinal cord transection in axolotl, by repressing expression of the mesodermal marker *brachyury*. Furthermore, we uncover other genes in the miR-200 pathway and provide evidence that, depending on the injury context, such as spinal cord lesion repair or spinal cord outgrowth during tail regeneration, miR-200a plays an important role in determining the identity of NSCs in the spinal cord during the regenerative process.

## RESULTS

### Transcriptional profiling identifies conserved miR-200a targets in homeostatic versus regenerating spinal cords

**Fig. 1.**
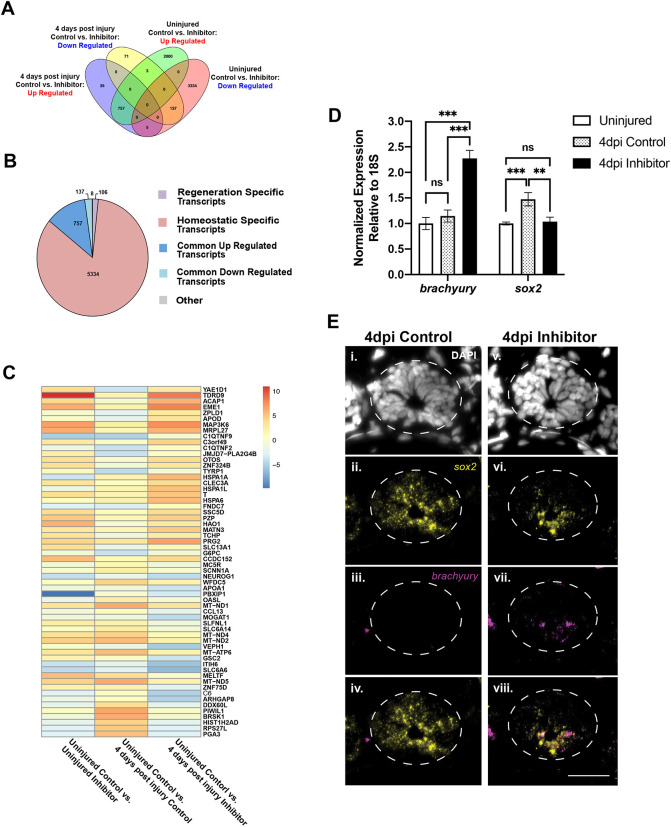
**miR-200a inhibition during spinal cord injury leads to *brachyury* expression in axolotl spinal cord stem cells.** (A) RNA-sequencing analysis identified a large subset of differentially regulated genes following injury. The Venn diagram compares the number of overlapping differentially expressed genes between uninjured, 4 days post-injury (dpi) control and miR-200a inhibitor-treated samples. (B) Pie chart showing the relative proportions of all transcripts that are differentially regulated. Regeneration-specific transcripts are defined as differentially expressed transcripts with a log2fc>1 or <1 and *P*adj<0.05 between 4 dpi control animals and 4 dpi animals treated with mir-200a inhibitor that were not differentially expressed in uninjured animals. (C) Log2fold change heat map demonstrates the 30 most up- and downregulated genes in uninjured and 4 dpi control versus miR-200a inhibitor-treated spinal cords. This analysis revealed that the transcription factor *brachyury* (*T*) is dramatically upregulated after miR-200a inhibition. (D) qRT-PCR analysis confirmed that miR-200a inhibition led to increased *brachyury* expression and blocked the upregulation of the neural stem cell marker *sox2* in 4 dpi spinal cords (*n*=3). (E) Fluorescent *in situ* hybridization confirmed the qRT-PCR analysis and revealed miR-200a inhibition leads to *brachyury* expression (*n*=5) in spinal cord cells and a failure to upregulate *sox2* expression (*n*=3). **P≤0.01, ***P≤0.001 (one-way ANOVA). ns is not significant. Data are mean±s.d. Scale bar: 50 µm.

Recently, miR-200a was identified as a key microRNA (miRNA) that inhibits *c-jun* expression in neural stem cells (NSCs) in the spinal cord after injury, and hence plays an important role in preventing reactive gliosis and promoting a pro-regenerative response (Sabin et al., 2019). To further elucidate the role of miR-200a in spinal cord regeneration and to identify additional mRNA targets for miR-200a, we performed additional RNA-sequencing (RNA-seq) analyses on uninjured and 4 days post-injury spinal cord tissue electroporated with a control or targeted antisense miR-200a inhibitor (Fig. 1A, Table S1). During normal regeneration at 4 days post-injury, there were 1163 genes that are differentially expressed (Log2 fold change ≥2-fold, *P*≤0.05) compared with uninjured spinal cords. Inhibition of miR-200a in the uninjured spinal cord resulted in 6235 transcripts with a greater than twofold differential expression compared with control uninjured spinal cords. Interestingly, of the 6235 differentially expressed genes, only 2760 were upregulated (Fig. 1A, Fig. S1, Table S1). We used GOrilla analysis to identify gene ontology (GO) terms for the subset of genes that were significantly upregulated after miR-200a inhibition. GO terms involved with translation, RNA metabolism, peptide metabolism and translation initiation were significantly enriched in this geneset (*P*≤10^−24^) (Fig. S1). Interestingly, the 3475 genes that were significantly downregulated in uninjured spinal cords after miR-200a inhibition were enriched for GO terms involved with organismal development, developmental process, cellular differentiation and signaling (*P*≤10^−22^) (Fig. S1).

Analysis of differentially expressed genes at 4 days post-injury after miR-200a inhibition identified a total of 1007 genes that were differentially expressed compared with control spinal cords. This is a much smaller geneset and suggests more-specific genes are regulated by miR-200a during spinal cord regeneration. A total of 797 genes were upregulated and 210 genes were downregulated after miR-200a inhibition (Fig. S1). Genes that were upregulated were enriched for GO terms involved with nucleic acid metabolism, specifically RNA metabolism and protein localization (*P*≤10^−6^) (Fig. S1). Interestingly, the top GO terms enriched in downregulated genes were involved with nervous system processes, specifically synaptic signaling and chemical synaptic signaling, as well as nervous system development (*P*≤10^−6^).

Taking a more-targeted gene-level approach, we generated a heat map of the 30 most significantly upregulated and downregulated genes in all four conditions (Fig. 1C). Consistent with the GO analysis, genes involved in RNA processing, nucleic acid metabolism and protein targeting were among the most upregulated genes in our dataset (*tdrd9*, *acap1*, *eme1* and *zfp324b*). Similarly, genes involved with neurotransmitter transport, neuronal polarization, neurotrophin signaling and neuronal differentiation were among the most downregulated genes (*slc6a6*, *brsk1*, *slc6a14*, *arhgap8* and *neurog1*). Surprisingly, the transcription factor *brachyury* (*T*) was among one of the most upregulated genes at 4 days post-injury in response to miR-200a inhibition (Fig. 1C). In 4 days post-injury controls, *brachyury* was not significantly upregulated in response to injury, the RNA-seq transcripts per million (TPM) values on control uninjured were 0.782 TPM versus 0.92 TPM for 4 days post-injury (Table S1). However, inhibition of miR-200a in uninjured spinal cords led to a twofold increase in *brachyury* expression (2.236 TPM), while the combination of miR-200a inhibition during injury led to a highly significant sevenfold increase in *brachyury* mRNA levels (5.8 TPM, Table S1).

We used quantitative RT-PCR (qRT-PCR) to verify genes of interest revealed by RNA-seq; this approach confirmed that *brachyury* is detectable at very low levels in uninjured and control 4 days post-injury spinal cords, but is significantly upregulated after miR-200a inhibition in 4 days post-injury spinal cords (Fig. 1D). This is an intriguing finding, as *brachyury* is considered a classical marker of mesodermal tissue and was originally thought to be absent from the nervous system. However, more recent research has identified a bipotent cell population during development, in which some spinal cord neural progenitor cells are developmentally derived from Sox2^+^/brachyury^+^ neuromesodermal progenitors (NMPs) (Garriock et al., 2015; Tzouanacou et al., 2009; Wymeersch et al., 2016). In the axolotl, the bona fide stem cells that line the central canal are identified by the expression of the glial cell marker GFAP and the neural stem cell marker Sox2. These GFAP^+^/Sox2^+^ cells respond to the injury, divide, migrate and repair a lesion in the spinal cord, or regenerate lost cells and tissues in the context of whole-tail regeneration (Sabin et al., 2015; Fei et al., 2014; Echeverri and Tanaka, 2002, 2003; McHedlishvili et al., 2007, 2012). Given that NMPs and axolotl glial cells both express Sox2 and that *sox2* is a miR-200a target during mouse brain development (Peng et al., 2012), we assayed *sox2* transcript abundance. Interestingly, although *sox2* is slightly upregulated in control 4 days post-injury compared with uninjured spinal cords, *sox2* expression did not increase in miR-200a inhibitor-treated spinal cords. Instead, the *sox2* transcript abundance remains near uninjured homeostatic levels (Fig. 1D). This observation suggests that axolotl *sox2* is not a direct target of miR-200a as it is in mammals (Pandey et al., 2015; Peng et al., 2012; Wang et al., 2013).

To identify the cells that express *brachyury* in the 4 days post-injury spinal cord after miR-200a inhibition, *in situ* hybridization was used. Cells lining the central canal are *brachyury*^+^ after miR-200a inhibition (Fig. 1Evii) and, importantly, this is the same population of cells that express *sox2* (Fig. 1Evi,viii). Collectively, these data indicate that miR-200a inhibition leads to increased *brachyury* expression in stem cells in the axolotl spinal cord. Although these progenitor cells have been traditionally thought of as NSCs due to their expression of the classical NSC marker *sox2*, they also express low levels of the mesodermal marker *brachyury* (Fig. 1D), suggesting that they are a population of bipotent stem cells and could have broader differentiation potential.

### Inhibition of miR-200a leads to changes in cell fate after spinal cord injury

**Fig. 2.**
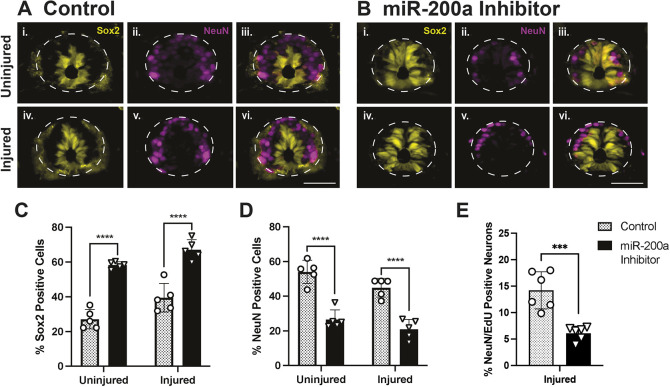
**Chronic miR-200a inhibition affects the birth of new neurons.** (A,B) Representative images of Sox2 and NeuN staining in uninjured and injured spinal cords injected with control (A) or miR200a inhibitor (B). (C) Inhibition of miR-200a for 2 weeks significantly increases the proportion of Sox2^+^ stem cells in the spinal cord throughout the regeneration zone (*n*=5). (D) Inhibition of miR-200a for 2 weeks also significantly reduces the proportion of NeuN^+^ cells in the spinal cord throughout the regeneration zone (*n*=5). (E) Moreover, miR-200a inhibition also results in a smaller proportion of newborn neurons compared with controls (*n*=6). ***P≤0.001, ****P≤0.0001 (two-way ANOVA). Data are mean±s.d. Scale bars: 50 µm.

To test the impact of miR-200a inhibition and subsequent upregulation of the mesodermal marker *brachyury* on the number of NSCs or neurons in the regenerating spinal cord, we quantified the number of Sox2^+^ NSCs and NeuN^+^ neurons in control versus inhibitor-treated animals (Fig. 2A,B). miR-200a inhibition was achieved using a specific antisense inhibitor that we have previously shown to significantly reduce miR-200a levels in the axolotl (Sabin et al., 2019). As previous work has shown that NSCs residing within 500 µm of the injury site partake in spinal cord regeneration (Sabin et al., 2015), we quantified the number of Sox2^+^ and NeuN^+^ cells 500 µm rostral and caudal to the injury site. This is the cell population known to be responsible for regeneration after injury and co-express GFAP and Sox2 (Fig. S2). Specifically, we quantified Sox2 and NeuN cells at 2 weeks post-injury, when Sox2^+^ NSCs have replenished the GFAP^+^ cell population and differentiated into new neurons under normal conditions (Albors et al., 2015; Echeverri and Tanaka, 2002, 2003; McHedlishvili et al., 2007, 2012). We discovered that injection with the miR-200a inhibitor resulted in a significant increase in the proportion of Sox2^+^ NSCs in the spinal cord in both uninjured and injured tissue (Fig. 2C). Moreover, injection with the miR-200a inhibitor also significantly reduced the proportion of neurons in both uninjured and injured tissue (Fig. 2D). To determine whether the number of newborn neurons is affected by miR-200a inhibition, we quantified the number of NeuN^+^ and EdU^+^ cells. We found that, overall, significantly fewer NeuN^+^/EdU^+^ cells were found in the miR-200a inhibitor animals (Fig. 2E). Collectively, these data demonstrate that the proportion of Sox2^+^ spinal cord stem cells increases in miR-200a inhibited animals, and fewer NeuN^+^ cells are found in the inhibitor-treated animals.

**Fig. 3.**
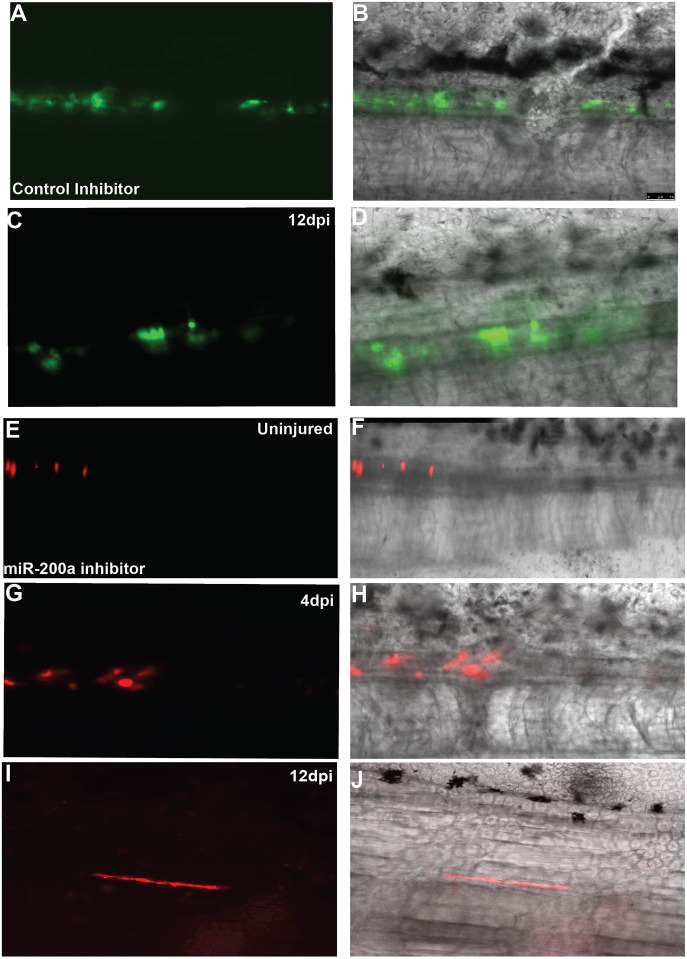
**miR-200a-inhibited spinal cord cells form muscle during spinal cord lesion repair.** (A-J) The cells lining the central canal of the spinal cord were labeled using GFAP promoter driving GFP or tdTomato by injection and electroporation. (A-D) Control cells were followed over a 14-day period and cells gave rise to new glial cells or neurons only (*n*=20). (E-J) Cells that were injected with the miR-200a inhibitor were followed in parallel over the same time period and were found to exit the spinal cord and give rise to muscle cells (*n*=25). Scale bar: 50 µm.

These findings suggest that after miR-200a inhibition, either more cells remain in a progenitor-like state or the increase in *brachyury* expression changes the fate of the cells. To address this issue, we used *in vivo* cell tracking to determine the fate of these cells during regeneration of the lesioned spinal cord. Previous work tracking the fate of GFAP^+^ spinal cord stem cells during regeneration of the lesioned spinal cord found that these cells proliferate and migrate to replace the region of injured neural tube, and that this is a bidirectional process (Sabin et al., 2015). The same technique was used in these studies; the axolotl GFAP promoter driving expression of a fluorescent protein was injected into the lumen of the spinal cord, and the animals were electroporated to label small groups of cells. The miR-200a inhibitor was injected into animals with fluorescently labeled cells and then the spinal cord ablation was performed (Sabin et al., 2019). The animals were imaged every 3 days over a 2-week time period. In the control labeled animals, we found the cells behaved as previously described, the labeled cells proliferated and partook in repair of the neural tube, replenished the endogenous stem cell population and differentiated to replace lost neurons (Fig. 3A-F) (Sabin et al., 2015). In contrast, in the miR-200a inhibitor-treated animals, although the cells proliferated and partook in repair of the lesioned spinal cord, we also discovered that the cells exited from the spinal cord and differentiated into muscle cells. The labeled cells that started in the spinal cord were always found in the muscle layer adjacent or directly above the spinal cord (Fig. 3K,L). In all miR-200a inhibitor-treated animals, we observed at least one muscle fiber being formed in all animals (*n*=25), although in some animals multiple fibers were seen. The muscle cell identity was confirmed by fixing some animals and performing immunofluorescence staining using an antibody against myosin heavy chain, which is specifically expressed in muscle (Fig. S3). Additionally, in inhibitor and control animals, some cells differentiated into neurons and remained within the neural tube to give rise to new glial cells (Fig. S7). These data suggest that miR-200a represses *brachyury* in *sox2*^+^ spinal cord stem cells, maintaining the cells in neural primed state. Inhibition of miR-200a in these cells results in the co-expression of neural (*sox2*) and mesoderm (*brachyury*) markers, converting the cells into a bipotent progenitor population capable of making both neural and mesodermal cells.

### Molecular regulation of progenitor cells by miR-200a

Our data indicate that inhibition of miR-200a leads to the expression of *brachyury* in stem cells within the axolotl spinal cord (Fig. 1D,E). However, the signaling pathway(s) upstream of *brachyury* expression are not known. As a first step, we first tested whether miR-200a could directly repress *brachyury* expression. The axolotl *brachyury* 3′ untranslated region (UTR) contains three miR-200a seed sequences; this indicates that miR-200a could directly regulate *brachyury* expression. Consistent with this hypothesis, co-transfection of B35 neural cells with a *brachyury* 3′ UTR luciferase reporter and miR-200a mimic led to decreased luciferase activity compared with the control mimic (Fig. S4A). This finding confirmed that miR-200a directly represses *brachyury* expression in axolotl spinal cord stem cells in homeostatic conditions and during normal regeneration.

**Fig. 4.**
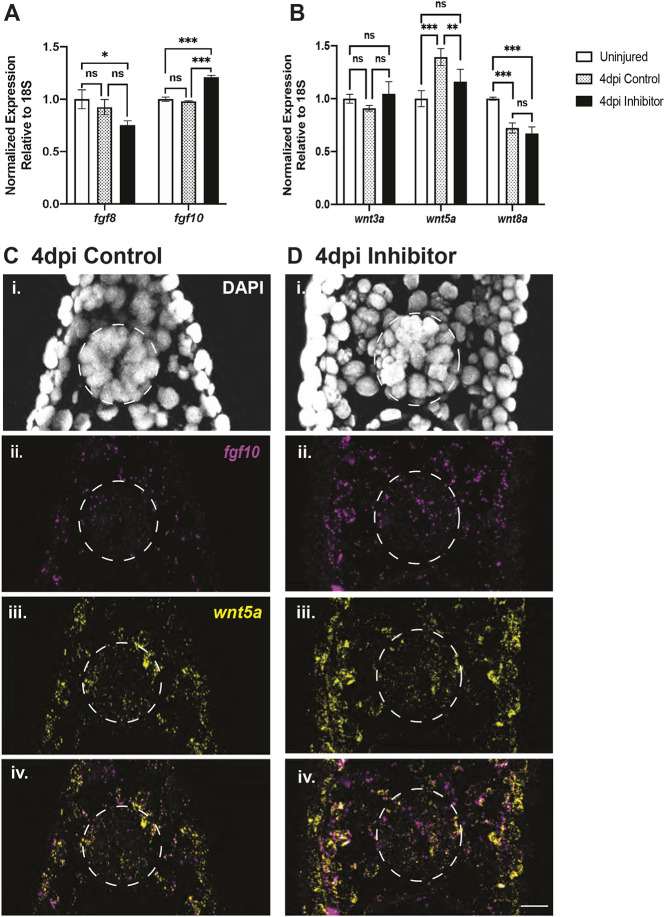
**miR-200a inhibition affects the expression of Wnt and FGF signaling ligands.** (A) qRT-PCR analysis revealed *fgf10*, but not *fgf8*, was significantly upregulated after miR-200a inhibition (*n*=3). (B) qRT-PCR analysis showed that miR-200a inhibition differentially affected the expression of *wnt5a*, but not additional Wnt ligands (*wnt3a* and *wnt8a*; *n*=3). (C,D) Fluorescent *in situ* hybridization in control (C) and miR-200a inhibitor-treated (D) animals confirmed the qRT-PCR analysis, and demonstrated an increase in *fgf10* expression and a downregulation of *wnt5a* within stem cells in the spinal cord (*n*=6). *P≤0.05, **P≤0.01, *** *P*≤0.001 (one-way ANOVA). ns is not significant. Data are mean±s.d. Scale bar: 50 µm.

During normal spinal cord regeneration in the context of a tail amputation model, it has been found that Wnt genes are re-expressed in the caudal 500 µm of the outgrowing spinal cord and are necessary for this outgrowth (Albors et al., 2015). Further studies have shown that inhibition of all Wnt or Fgf signaling after tail amputation abolishes regenerative outgrowth, suggesting both are necessary for spinal cord and tail regeneration (Ponomareva et al., 2015). As both Fgf and Wnt signaling regulate cell fate decisions of *brachyury*^+^/*sox2*^+^ NMPs during development, we first tested whether Fgf signaling could be affected by miR-200a inhibition during regeneration. We measured expression of *fgf8* and *fgf10* by qRT-PCR on isolated spinal cord tissue. *fgf8* expression was not significantly downregulated at 4 days post-injury after miR-200a inhibition compared with uninjured spinal cords (Fig. 4A), whereas *fgf10* expression was significantly upregulated after miR-200a inhibition in 4 days post-injury spinal cords compared with controls (Fig. 4A). This finding is consistent with the idea that miR-200a inhibition could lead to an increase in Fgf ligand expression in regenerating spinal cords. However, given that Wnt signaling directly regulates Brachyury expression (Arnold et al., 2000; Yamaguchi et al., 1999) and NMP cell fate decisions (Bouldin et al., 2015; Garriock et al., 2015; Martin, 2016; Martin and Kimelman, 2008), we wanted to further examine the role of Wnt signaling.

The expression levels of *wnt3a*, *wnt5a* and *wnt8a* were quantified using qRT-PCR (Fig. 4B), as these Wnt ligands have been associated with maintaining NMP fate decisions. Both *wnt3a* and *wnt8* transcript levels were not significantly altered in the inhibitor-treated animals compared with controls. However, we did detect a significant difference in *wnt5a* levels. In 4 days post-injury controls, *wnt5a* was upregulated after injury, although this change in expression was not found in the miR-200a inhibitor-treated animals. To further verify the qRT-PCR results for Fgf and Wnt genes, we performed fluorescent *in situ* hybridization for *fgf10* and *wnt5*a in control and miR-200a inhibitor-treated regenerating animals. This confirmed that, indeed, *fgf10* transcript levels are upregulated in cells within the spinal cord in comparison with the control regenerating animals (Fig. 4C,D). Interestingly, *in situ* hybridization appeared to demonstrate an increase in *wnt5a* expression outside of the spinal cord in inhibitor-treated samples. However, *wnt5a* transcript levels were downregulated in cells within the spinal cord in comparison with controls (Fig. 4C,D), which was confirmed through fluorescent quantification (Fig. S4B). To better determine the expression dynamics of both *fgf10* and *wnt5a*, we further quantified the proportion of cells within the spinal cord containing either *fgf10* or *wnt5a*. We discovered that the number of *fgf10^+^* cells significantly increased in inhibitor-treated animals compared with controls. In contrast, although most cells within the spinal cord contain *wnt5a*, no significant difference was detected in the number of *wnt5a*^+^ cells between control or inhibitor-treated samples (Fig. S4C).

Although here we see only changes in *wnt5a* expression, a Wnt that is known to play an important role in regeneration (Albors et al., 2015), there are many additional Wnt ligands; therefore, Wnt signaling activity could still be affected by miR-200a inhibition. To establish a baseline for Wnt signaling activity after spinal cord injury, we assayed *lef1* expression, which is a direct transcriptional target downstream of Wnt signaling (Filali et al., 2002). *lef1* expression was significantly upregulated in control 4 days post-injury compared with uninjured spinal cords (Fig. S5A), indicating a potential increase in Wnt signaling after injury. Remarkably, *lef1* expression was significantly upregulated even further after miR-200a inhibition in 4 days post-injury compared with control regenerating spinal cords (Fig. S5A). Collectively, these data indicate that miR-200a inhibition could result in increased Wnt signaling, potentially independently of changes in Wnt ligand expression.

### miR-200a modulates Wnt signaling activity by directly targeting β-catenin

Although miR-200a inhibition could lead to increased Wnt signaling, it was not clear how this was occurring. During tumor progression, miR-200a inhibits the epithelial-to-mesenchymal transition subsequently blocking tumor cell metastasis (Su et al., 2012; Zaravinos, 2015). This is partially achieved through the direct repression of β-catenin (*ctnnb1*) by miR-200a, resulting in decreased Wnt signaling (Su et al., 2012). We did not observe a significant upregulation of a specific Wnt ligand after miR-200a inhibition in the spinal cord cells (Fig. 4B). However, as determined by *lef1* expression, miR-200a inhibition could lead to increased Wnt signaling (Fig. S5A). Therefore, we hypothesized that miR-200a might regulate Wnt signaling by targeting *ctnnb1*. To test our hypothesis, we first assayed for changes in *ctnnb1* abundance. qRT-PCR analysis confirmed that, after injury in control 4 days post-injury spinal cords, there is an increase in *ctnnb1* abundance compared with uninjured spinal cords, similar to what we observed for *lef1* (Fig. S5A). There is a slight increase of *ctnnb1* transcript levels after miR-200a inhibition compared with control 4 days post-injury spinal cords (Fig. S5A), indicating *ctnnb1* could be a direct target of miR-200a in axolotl.

To determine whether miR-200a could target axolotl *ctnnb1*, we cloned the *ctnnb1* 3′ UTR and identified two miR-200a seed sequences. We subcloned the *ctnnb1* 3′ UTR into a luciferase reporter and co-transfected cells with a control mimic or miR-200a specific mimic. There was decreased luciferase activity in miR-200a mimic transfected cells compared with control, suggesting that miR-200a could regulate *ctnnb1* expression (Fig. S5B). To confirm that the decrease in luciferase activity is due to direct regulation by miR-200a, we mutated both seed sequences in the *ctnnb1* 3′ UTR and repeated the luciferase experiments. Mutation of the miR-200a seed sequences completely alleviated the repression, confirming that axolotl *ctnnb1* is a direct target of miR-200a, similar to mammals (Fig. S5B).

Taken together, these data are consistent with the idea that miR-200a could modulate Wnt signaling through the direct regulation of *ctnnb1* levels. Inhibition of miR-200a leads to increased *lef1* expression, which is indicative of increased Wnt signaling. Increased levels of Wnt signaling may contribute to the increased *brachyury* expression and changes in *fgf10* levels in axolotl stem cells after spinal cord lesion.

### The role of spinal cord stem cells in spinal cord injury versus tail regeneration

**Fig. 5.**
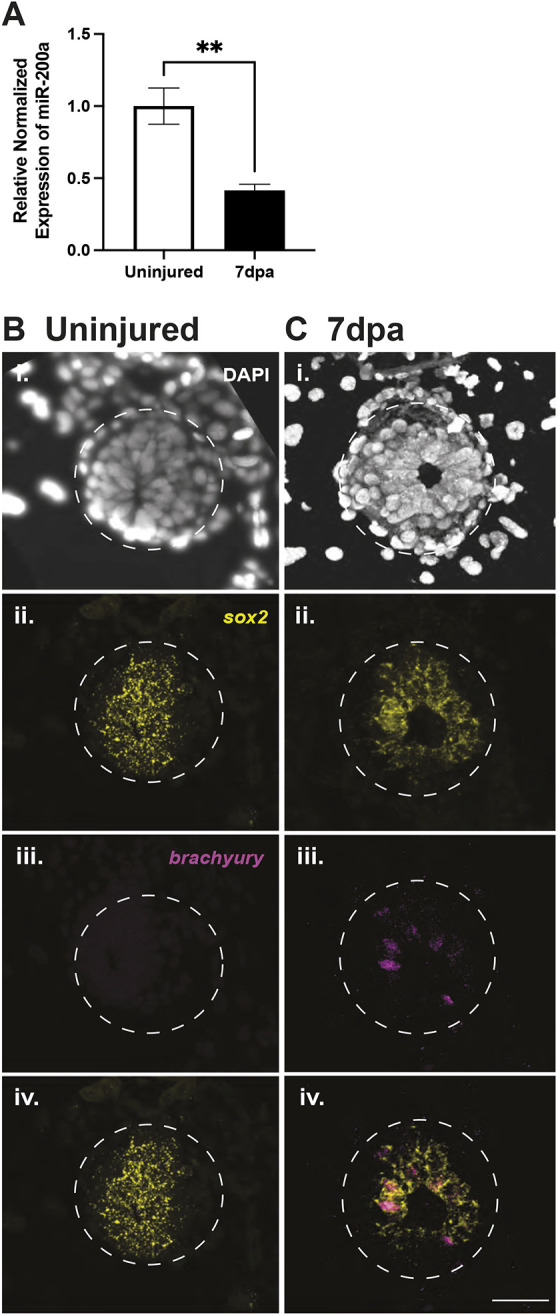
**Spinal cord amputation leads to *brachyury* expression in spinal cord stem cells.** (A) qRT-PCR demonstrates that miR-200a is significantly reduced in the adult spinal cord at 7 days post-tail amputation (dpa) in comparison with uninjured spinal cord tissue (**P≤0.01, unpaired *t*-test; *n*=3). (B) Fluorescent *in situ* hybridization revealed that *sox2* is expressed within the uninjured adult spinal cord, while *brachyury* is absent (*n*=2). (C) At 7 days post-amputation, *brachyury* was localized to spinal cord stem cells that share an overlapping expression pattern with *sox2* (*n*=2). Scale bar: 50 µm.

**Fig. 6.**
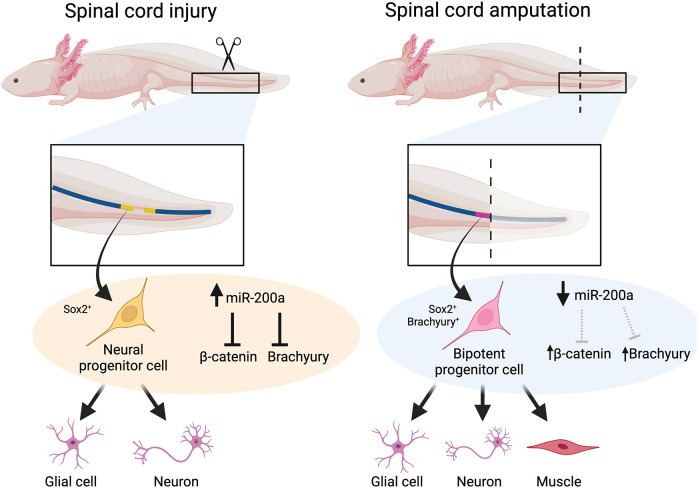
**A proposed model for the role that miR-200a plays in different injury paradigms.** (Left) When a lesion occurs in the spinal cord, miR-200a levels remain high, which inhibits *brachyury* expression and modifies levels of β-catenin, potentially stabilizing a neural stem cell identity in the cells adjacent to the injury site. After spinal cord injury, these cells replace neurons and glial only. (Right) In contrast, when the tail is amputated, progenitor cells respond to injury cues and replace multiple cell types of different developmental origins. These cells in the spinal cord then upregulate *brachyury* in the *sox2*^+^ stem cells of the spinal cord and direct these cells to proliferate and form cells of both ectodermal and mesodermal origin.

We have shown that when a lesion occurs in the axolotl spinal cord, the glial cells adjacent to the injury site respond to the injury cue and proceed to behave like NSCs; they divide, migrate, self-renew and replace lost neurons. However, previous work has shown that during spinal cord regeneration after tail amputation, rather than injury, these glial cells can transdifferentiate and give rise to cells of both the ectodermal and mesodermal lineage (Echeverri and Tanaka, 2002; McHedlishvili et al., 2007). We next examined the expression of *brachyury* in the context of whole tail regeneration and discovered that it is expressed in the *sox2^+^* stem cells of the spinal cord 500 µm adjacent to the injury site at 4 days post-amputation (Fig. S6). To determine whether this is an attribute of the larval animals only, we also examined regenerating tail tissue from 2-year-old adult animals. Using qRT-PCR, we discovered that miR-200a is significantly reduced at 7 days post-tail amputation in adult animals (Fig. 5A). Moreover, *in situ* hybridization revealed that the progenitor cells in the adult spinal cord also co-express *brachyury* and *sox2* during tail regeneration (Fig. 5B,C). These data suggest that, during spinal cord regeneration, the cells lining the central canal determine what tissue types need to be restored. When only a small region of the neural tube needs to be regenerated following injury, the progenitor cells adopt a NSC state to successfully regenerate the spinal cord. During whole-tail regeneration following amputation, when multiple tissue lineages must be regenerated, these cells within the spinal cord become bipotent progenitors capable of making mesoderm and ectoderm (Fig. 6). Collectively, these experiments have shed light on the context-dependent nature of miRNA signaling during spinal cord lesion repair versus tail amputation, and have identified new signaling pathways that regulate progenitor cell fate during axolotl spinal cord regeneration.

## DISCUSSION

The current study has identified miR-200a as a regulator of stem cell fate in the regenerating axolotl spinal cord. GO term analysis of genes downregulated in the uninjured and 4 days post-injury spinal cord after miR-200a inhibition showed that these genes were involved with nervous system development, organismal development, synaptic signaling and cellular differentiation (Fig. 1, Fig. S1). Specifically, genes involved with neuronal differentiation (*neurog1* and *neurod4*) and neuronal processes like synaptic transmission (*chrnb1* and *gabra4*) and neurotransmitter uptake (*slc6a6*, *slc18a3* and *slc6a14*) were downregulated (Fig. 1, Fig. S1). This suggests that miR-200a normally functions to promote NSC identity. This is consistent with multiple reports across various species that inhibition of miR-200a and other miR-200 family members results in the loss of neural progenitor identity and precocious neuronal or glial differentiation (Buller et al., 2012; Choi et al., 2008; Morante et al., 2013; Peng et al., 2012; Trumbach and Prakash, 2015; Vallejo et al., 2011). However, we have found that, in the axolotl spinal cord, even in uninjured conditions in larval or adult axolotls, the cells lining the central canal express low levels of *brachyury* and *sox2*, the classical markers of mesoderm and neural stem cells (Fig. 1D, Fig. 5). These cells may represent a bipotent progenitor cell population and our data suggest that increased levels of *brachyury* are necessary for a progenitor to make the decision to exit the spinal cord and become a cell type of mesodermal origin (Fig. 6).

During embryonic development in multiple species, a small population of cells that co-expresses Sox2 and brachyury have been identified and are now called neuromesodermal progenitor cells (Gouti et al., 2014; Henrique et al., 2015; Jurberg et al., 2013; Kimelman, 2016b; Taniguchi et al., 2017; Tsakiridis et al., 2014; Tsakiridis and Wilson, 2015; Turner et al., 2014; Tzouanacou et al., 2009). Neuromesodermal progenitor cell commitment to the neural lineage is partially determined by the relative levels of Sox2 compared with brachyury, given that the two transcription factors function to antagonize one another (Koch et al., 2017), and by the respective levels of Fgf versus Wnt that the progenitor cells encounter (Goto et al., 2017; Row et al., 2018; Turner et al., 2014). To date, a definitive population of neuromesodermal progenitors has not been defined during axolotl development; however, work published by Taniguchi et al. has shown that a posterior region of the axolotl neural plate is positive for *brachyury* and *sox2*, and that this region gives rise to mesoderm during development (Taniguchi et al., 2017). This finding is consistent with the idea that the axolotl may also have a bipotent progenitor pool of cells established during early development; however, more work is needed, especially lineage tracing to establish whether the behavior of these cells is similar to their behavior in other species such as chick, mouse and zebrafish. The results we obtained using qRT-PCR and RNAscope *in situ* show that *brachyury* and *sox2* are detected in the progenitor cells of the spinal cord in both larval and adult axolotls, and suggest that axolotls retain a population of cells in the spinal cord throughout life that are bipotent. Work from McHedlishvili et al. previously showed that adult axolotl retains expression of embryonic markers of dorsal/ventral patterning, e.g. *pax7*, *pax6* and *shh* genes, that are not expressed in adult mammalian spinal cord (McHedlishvili et al., 2007). They additionally showed that, like earlier lineage tracing work in axolotl, cells from the spinal cord do in fact migrate out and form a range of other cell types, including blood vessels, skin, cartilage and muscle cells. Overall, these bodies of work indicate that the cells in the axolotl spinal cord retain a multi-potent progenitor cell state and are capable of responding to injury cues that direct them towards different cell fates as needed. Very early work on tail regeneration in salamanders had already hinted that the terminal vesicle structure formed at the growing end of the spinal cord during tail regeneration was an area of epithelial-to-mesenchymal transition where cells delaminate from the neural tube and exit to contribute to regeneration of surrounding tissues of other developmental lineages (Benraiss et al., 1997; Egar and Singer, 1972; O'Hara et al., 1992). Our data now provides molecular insight into the identity of these cells. We found that miR-200a-inhibited cells increase levels of *brachyury* and then, during regeneration of a spinal cord lesion, form muscle that is not observed in control regenerating lesions. However, we have not observed that these cells form cartilage, skin, fin mesenchyme or any other cell type. We cannot rule out the possibility that they have this potential, but lineage tracing is limited because the fluorescent protein expression is driven by the GFAP promoter, which we expect is turned off as the cells differentiate, and because imaging every 3 days means some differentiation events might be missed. We observed in all animals where miR-200a is inhibited that at least one muscle fiber is formed from the labeled cells; however, some differentiation events might be missed owing to the limitations of our labeling technique.

We also found that, in uninjured miR-200a inhibitor-treated animals, we see an increase in Sox2-positive cells and a decrease in NeuN-positive cells, as seen in injured miR-200a inhibitor-treated animals (Fig. 2B,C). This suggests that miR-200a plays a role in homeostatic maintenance of the neural stem cell population and in potentially directing these cells towards differentiation to neurons. In the future, it will be interesting to determine whether members of the miR-200 family play a role in the development of the spinal cord in axolotls. However, here we have focused on their role in regeneration and have found that miR-200a inhibition after injury specifically leads to upregulation of brachyury in the Sox2-positive neural stem cells.

It is still not clear whether brachyury directly regulates Sox2 levels in the regenerating axolotl spinal cord or whether it is via an indirect mechanism. Work from other labs in other research organisms has indicated that brachyury and Sox2 can have a mutually repressive relationship (Kimelman, 2016; Koch et al., 2017; Martin, 2016). We have shown that miR-200a directly regulates *brachyury* and *ctnnb1* via seed sequences in the 3′ UTR of these genes. When miR-200a is inhibited in the spinal cord cells, *brachyury* is expressed at higher levels in these cells, but Fgf and Wnt levels are also perturbed. Work in progress on NMPs has shown that feedback loops exist between *ctnnb1*, Fgf and Wnt genes, and hence a complex signaling network might exist that is driven by specific levels of certain regulators in these cells at particular times. An additional level of complexity is the fact that Wnt is a secreted protein and although we see its downregulation within the progenitor cells in the spinal cord, we also see that cells outside the spinal cord express Wnt (Fig. 4) and therefore the progenitor cells might also be influenced by external gradients of Wnt protein.

During development, Wnt and Fgf signaling tightly regulate neuromesodermal cell fate decisions (Goto et al., 2017; Gouti et al., 2015, 2017; Martin, 2016), and both proteins are known to play important roles in regeneration (Sun et al., 2002; Wilson et al., 2000; Zhang et al., 2000, 2002; Caubit et al., 1997; Ghosh et al., 2008; Lin and Slack, 2008; Stoick-Cooper et al., 2007; Tanaka and Weidinger, 2008; Wehner et al., 2017; Zakany and Duboule, 1993). Canonical Wnt signaling is crucial for radial glial cell proliferation during neural tube development (Shtutman et al., 1999) and for spinal cord regeneration in zebrafish (Briona et al., 2015). Therefore, it is not surprising to see a potential increase in Wnt signaling during spinal cord regeneration in axolotl. However, it is interesting that miR-200a does not regulate expression of Wnt ligands, but instead regulates *ctnnb1* levels (Fig. S5). This is reminiscent to the role of miR-200a in inhibiting EMT by repressing *ctnnb1* and canonical Wnt signaling (Su et al., 2012; Zaravinos, 2015). The increase in *ctnnb1* levels after miR-200a inhibition is not statistically significant; however, slight changes in transcript abundance can have profound effects on protein levels (Schwanhausser et al., 2011). Therefore, a modest increase in transcript abundance could represent a biologically significant increase in β-catenin protein levels.

The signals that inform injured cells what tissue must be replaced remain a mystery. Here we show that glial cells in the spinal cord appear to sense the difference between a lesion of the spinal cord that primarily needs replacement of neural stem cells and neurons, versus regeneration in the context of whole tail regeneration where cells of multiple developmental germ layer origin must be regenerated. Interestingly we find that cells of both the larval and adult tail regenerate bipotent progenitors that express *brachyury* and *sox2* in response to tail amputation, suggesting that the presence of these bipotent progenitors is not only a hallmark of embryonic development, but rather a stem cell population that is maintained in the animals specifically for regeneration. In the future it will be important to determine if all cells in the spinal cord have this potential or whether there are sub-populations of stem cells present in the axolotl spinal cord.

## MATERIALS AND METHODS

### Animal handling and spinal cord injury

All axolotls used in these experiments were obtained and bred at the University of Minnesota or the Marine Biological Laboratory in accordance with IACUAC regulations. Prior to all *in vivo* experiments, animals (3-5 cm) were anesthetized in 0.01% *P*-amino benzocaine (Sigma). Spinal cord ablations were performed as previously described (Diaz Quiroz et al., 2014; Sabin et al., 2015). Briefly, a 26-gauge needle was used to clear away skin and muscle to expose the spinal cord 6-10 muscle bundles caudle to the cloaca. Then, using the needle, a segment of spinal cord one muscle bundle thick, ∼500 μm, was removed. Animals were placed in cups and monitored for the duration of the experiments.

### Immunohistochemistry and EdU

Tissue was harvested and fixed in fresh 4% paraformaldehyde (Sigma) overnight at 4°C. Tails were then washed three times in phosphate-buffered saline+0.1% Tween 20 (PBSTw). Next, the tails were incubated in a 50:50 solution of PBSTw and 30% sucrose. Finally, tails were transferred to 30% sucrose solution and allowed to equilibrate overnight at 4°C. The next day samples were embedded for cross-sectioning in TissueTek (Sakura) and stored at −20°C.

For EdU staining, animals were injected intraperitoneal with EdU at a concentration of 0.5 μg/μl in PBS+1% Fast Green at 5 and 7 days post-injury then harvested at 14 days post-injury. The tissue was processed for sectioning as described above and stained using the Click-iT EdU Imaging Kit (Invitrogen) according to the manufacturer's instructions.

After staining for EdU, samples were processed for immunohistochemical analysis using either anti-Sox2 (1:100, ab97959, Abcam) or anti-NeuN (1:100, MAB377, Chemicon) primary antibodies as previously described (Sabin et al., 2015, 2019). Briefly, slides were subjected to a boiling citrate antigen retrieval step for 10 min and then washed with PBSTw three times for 5 min each. Samples were blocked (PBS+0.1% Triton-X+2% bovine serum albumin +2% goat serum) for 1 h at room temperature then incubated overnight at 4°C in primary antibodies diluted in blocking buffer. The next day, slides were washed four times with PBSTw and then incubated with secondary antibody (1:200, A21235 and A11011, Invitrogen) diluted in blocking buffer for 2 h at room temperature and cell nuclei were counterstained with 4′,6-diamidino-2-phenylindole (DAPI) (1:10,000). After secondary antibody incubation, the slides were washed four times with PBSTw and mounted in Prolong Anti-fade mounting solution (Invitrogen). For Sox2 and GFAP immunostaining, the samples were similarly processed using an anti-GFAP (Chemicon, AB5804, 1:100) primary antibody. All samples were imaged using an inverted Leica DMI 6000B fluorescent microscope. All images were generated using Fiji and cells were counted with the Cell Counter plug-in.

### Quantitative reverse transcriptase polymerase chain reaction

Injured spinal cords 500 μm rostral and 300 μm caudal to the lesion from 7-10 control or miR-200a inhibitor electroporated animals were micro-dissected and pooled for each biological replicate. Total RNA was isolated using Trizol (Invitrogen) according to the manufacturer's instructions. Subsequent cDNA was synthesized from 1 μg of DNaseI (NEB) treated RNA using either High Capacity cDNA Reverse Transcription kit (Applied Biosystems) or miRCURY LNA RT Kit (Qiagen). The qRT-PCR was carried out using Light Cycler 480 SYBR Green I Master (Roche). MicroRNA qRT-PCR was carried out with custom designed LNA primers to conserved miRNAs using the miRCURY LNA miRNA PCR Assay kit (Qiagen) and custom primers from IDT were used to quantify axolotl mRNAs: *18S*_F, CGGCTTAATTTGACTCAACACG; *18S*_R, TTAGCATGCCAGAGTCTCGTTC; *brachyury*_F, GAAGTATGTCAACGGGGAAT; *brachyury*_R, TTGTTGGTGAGCTTGACTTT; *sox2*_F, TTGTGCAAAATGTGTTTCCA; *sox2*_R, CATGTTGCTTCGCTTTAGAA; *wnt3a*_F, AAGACATGCTGGTGGTCTCA; *wnt3a*_R, CCCGTACGCATTCTTGACAG; *wnt5a*_F, ACCCTGTTCAAATCCCGGAG; *wnt5a*_R, GGTCTTTGCCCCTTCTCCAA; *wnt8a*_F, TTGCTGTCAAATCAACCATG; *wnt8a*_R, TGCCTATATCCCTGAACTCT; *ctnnb1*_F, ACCTTACAGATCAAAGCCAG; *ctnnb1*_R, GGACAAGTGTTCCAAGAAGA; *lef1*_F, GTCCCACAACTCCTACCACA; *lef1*_R, TAGGGGTCGCTGTTCACATT; *fgf8*_F, TTTGTCCTCTGCATGCAAGC; *fgf8*_R, GTCTCGGCTCCTTTAATGCG; *fgf10*_F, AAACTGAAGGAGCGGATGGA; *fgf10*_R, TCGATCTGCATGGGAAGGAA.

### Fluorescent *in situ* hybridization

All RNAscope *in situ* hybridization procedures were performed according to the manufacturer's instructions (Advanced Cell Diagnostics). In brief, cryosections were incubated in PBS for 10 min to remove the OCT, and then baked at 60°C for 30 min. The slides were next post-fixed in 4% paraformaldehyde for 15 min at 4°C, and then dehydrated in a graded series of ethanol dilutions before being incubated in absolute ethanol for 5 min. After briefly air-drying the slides for 5 min, sections were next treated with hydrogen peroxide to quench endogenous peroxidase activity for 10 min at room temperature. Next, samples were briefly washed in deionized water, then incubated in target retrieval buffer at 90°C for 5 min. Following target retrieval, the slides were rinsed in deionized water for 15 s and treated with absolute ethanol for 3 min. Slides were next permeabilized in protease III for 30 min before hybridization with RNAscope probes at 40°C for 2 h. Following hybridization, sections were placed in 5× SSC overnight. The next day, sections were incubated in Amp1 and Amp2 at 40°C for 30 min each, followed by Amp3 for 15 min. Next, slides were treated with HRP-C1 to detect *brachyury* or *fgf10*, followed by a 30-min incubation in OpaI-690 fluorescent dye. After treatment with HRP-blocking buffer, samples were next incubated in HRP-C2 to detect either *sox2* or *wnt5a*, followed by a 30-min incubation in OpaI-570 dye. After an additional treatment with HRP blocking buffer, slides were counterstained with DAPI and imaged using a Zeiss 780 confocal microscope.

### Cell tracking

Cells of the uninjured spinal cord were transfected with a construct containing a GFP or tdTomato fluorescent protein under the control of the axolotl GFAP promoter. The cells were injected and electroporated as previously described (Echeverri and Tanaka, 2003; Sabin et al., 2015). One day after electroporation, the animals were screened for fluorescent cells. Positive animals were then injected with a control inhibitor or miR-200a inhibitor and then a spinal cord lesion performed as described by Sabin et al. (2019). Animals were imaged every 3 days until the lesion site was no longer visible and the animals regained motor and sensory function, typically 12-14 days post-injury.

### Whole-mount immunohistochemistry

Animals were fixed 14 days post-injury in 4% paraformaldehyde for 1 h at room temperature. The tail portion containing the labeled cells was trimmed and processed for wholemount immunostaining. Briefly, the tissue was washed three times for 5 min in PBS+0.1% Tween-20 (PBST) and then incubated in 0.2% Triton X100 for 10 min. The tissue was then blocked in 10% goat serum plus 1% BSA for 1 h at room temperature. Tissues were incubated in an anti-myosin (1:100, MF20, DSHB) monoclonal primary antibody diluted in blocking buffer overnight at 4°C. The next day, samples were washed 4×10 min at room temperature with PBST and then incubated in an anti-mouse-Alex-568 (1:200, Invitrogen) secondary antibody diluted into blocking buffer. Samples were washed 3×30 min with PBST, and then mounted in 80% glycerol and imaged on an inverted Leica DMI6000B.

### Cloning 3′ untranslated regions for miRNA luciferase assays

For 3′ UTR luciferase experiments, primers were designed to amplify the *brachyury* and *ctnnb1* 3′ UTR based off sequences obtained from axolotl-omics.org. All the 3′ UTRs were amplified with a 5′ SpeI and 3′ HindIII restriction site: *brachyury* 3′ UTR For 1, AGCACTAGTATGTGAAATGAGACTTCTAC; *brachyury* 3′ UTR Rev 1, TGCAAGCTTCTTATTCTTCCCATTTAACTTAAA; *ctnnb1* 3′ UTR For 1, ATAACTAGTTTGTGTAATTTTTCTTAGCTGTCATAT; *ctnnb1* 3′ UTR Rev 1, ATCAAGCTTAATTGCTTTATAGTCTCTGCAGAT; *ctnnb1* 3′ UTR SDM1 For, AGTGCCTGATGAATTCAACCAAGCTGAG; *ctnnb1* 3′ UTR SDM1 Rev, CTCAGCTTGGTTGAATTCATCAGGCACT; *ctnnb1* 3′ UTR SDM2 For, ATTTAATGGTGTAGGAATTCAATAGTATAA; *ctnnb1* 3′ UTR SDM2 Rev, TTATACTATTGAATTCCTACACCATTAAAT.

The PCR fragments and pMiR Report (Life Technologies) were digested with SpeI and HindIII (NEB), and the fragments were ligated overnight at 4°C with T4 DNA Ligase (NEB) and heat-shock transformed into DH5α competent *E. coli* (Promega).

### Mutation of miR-200 sites in brachyury 3′ UTR

To mutate the 3 miR-200a and 3 miR-200b sites in the axolotl Brachyury 3′ UTR, we used the QuikChange Lightening Multi Site-Directed Mutagenesis kit (Agilent) as per the manufacturer's instructions. The nucleotides used were as follows: miR-200a1 SDM, gactgctttctatggacactttttaatttctgaagataagctcccacccg; miR-200a2 SDM, cacacataaatcttttcgtgctgaacaaattatgatccatgaaaccagtgcatcattt; miR-200a3 SDM, tccaatgtgtgtaatcctctcaattatcgcctctgcgtgtagaatgtc; miR-200b1 SDM, atgcattacaatgcattgttttctggacggcaatgaaagctgtgatgaaatatttaagat; miR-200b2 SDM, caccataagagacaataaatgcaccggaatactgtgatatttgatgcctgcac; miR-200b3 SDM, gaatcattaccatgtatttatcaggccggaatattcaaaatgtgacttcctctgtga.

### 3′ UTR luciferase experiments

B35 neuroblastoma cells were plated in a 96-well plate (Celltreat Scientific Products) at a concentration of 2.0×10^5^ cells/ml and allowed to adhere overnight. The next day, cells were co-transfected with the appropriate Luciferase 3′ UTR reporter plasmid, β-Galactosidase control, and 100 nM of miR-200a, miR-200b or control mimic (Qiagen) per well using Lipofectamine 3000 (Invitrogen). After 48 h, luciferase activity was determined using Dual Light Luciferase & β-Galactosidase Reporter Gene Assay System (ThermoFisher Scientific) according to the manufacturer's protocol.

### Pie chart and Venn diagram generation

Pie charts were generated using previously published data (Sabin et al., 2019) to represent the total number of differentially expressed genes in a given comparison using Excel. Venn diagrams were generated with Venny (v2.1.0) (Oliveros 2007-2015) and saved as .csv files to be modified in Adobe Illustrator.

### Gene ontology analysis

Gene ontology (GO) terms were determined using GOrilla (Eden et al., 2009). We used two unranked lists of genes: a background list (all differentially expressed genes in our dataset) and a target list (genes that were differentially regulated in a given comparison). Using this approach, GOrilla generated a list of enriched biological process GO terms and we selected the top 9-13 terms with the lowest *P*-value and generated representative bar graphs using Excel.

### Calculation of the proportion and distribution of neural stem cells and newborn neurons

The number of Sox2^+^ neural stem cells were counted in control and miR-200a inhibitor spinal cords at 2 weeks post-injury. The proportion of Sox2^+^ neural stem cells was calculated as (total number of Sox2+ neural stem cells/the total number of DAPI+ spinal cord cells)×100. Similarly, the proportion of neurons was calculated as (total number of NeuN^+^ cells/the total number of DAPI^+^ cells)×100. To analyze regenerative neurogenesis, control or miR-200a inhibitor animals were injected with EdU at 5 and 7 days post-injury and tails were harvested for cryosectioning at 14 days post-injury. The proportion of newborn neurons was determined as (number of NeuN^+^/EdU^+^ double positive neurons/the total number of NeuN^+^ neurons)×100.

### Statistical analyses

All results are presented as mean±s.d. unless otherwise stated. Analyses were performed using Microsoft Excel or GraphPad Prism v9. Dataset means were compared using a one- or two-way ANOVA with a Tukey test (for multiple comparisons) or Dunnett test (to compare to a control mean). When two groups were compared, an unpaired *t-*test was used. When multiple comparisons were made using an unpaired *t*-test, an adjusted *P*-value was determined using the two stage Benjamin, Krieger and Yekutieli procedure with a false discovery rate <5%. Differences between groups were considered significant at four different levels: (*P≤0.05, **P≤0.01, ***P≤0.001 and ****P≤0.0001) and are indicated in the figure legends.

## Supplementary Material

Supplementary information
