# Supplementary material for: Regulation of stem cell identity by miR-200a during spinal cord regeneration
Source: Development. 2022 Feb 14;149(3):dev200033. doi: 10.1242/dev.200033 (PMC8918811; doi:10.1242/dev.200033)
Supplement: Supplementary information [file develop-149-200033-s1.pdf]

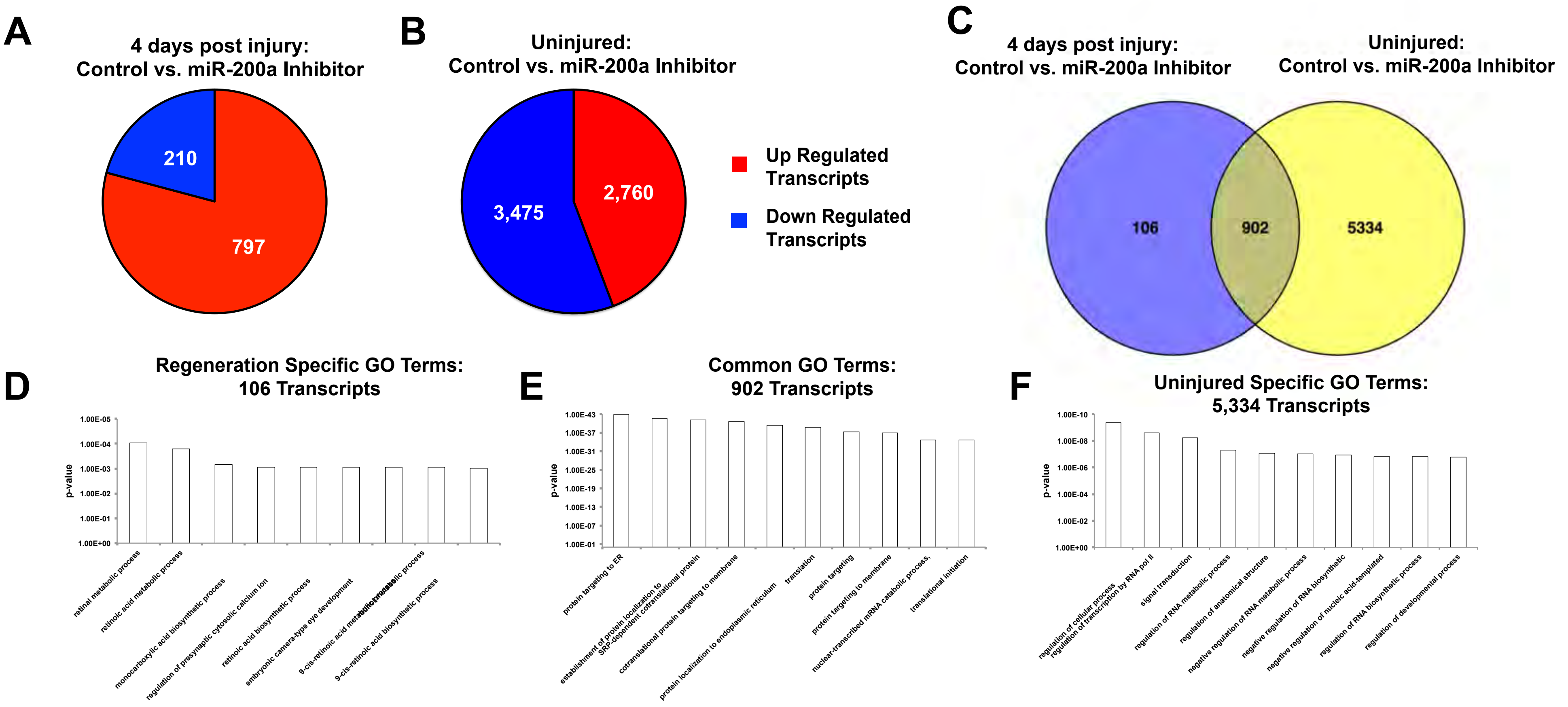

Fig. S1. miR-200a affects expression of common and unique gene sets in the uninjured and regenerating spinal cord. (A, B, C) Pie chart representation of the proportion of up-regulated (Red) or down-regulated (Blue) genes in (B) uninjured control compared to uninjured miR-200a inhibitor electroporated spinal cords or (A) 4 days post injury control compared to 4 days post injury miR-200a inhibitor electroporated spinal cords. (C). Pie chart illustrating the number of overlapping versus individual genes that are differentially regulated. (B-F) Gene Ontology terms enriched in gene specifically in (B) control regenerating or (E) genes common to all data sets or specific to uninjured tissue (F).

Development • Supplementary information

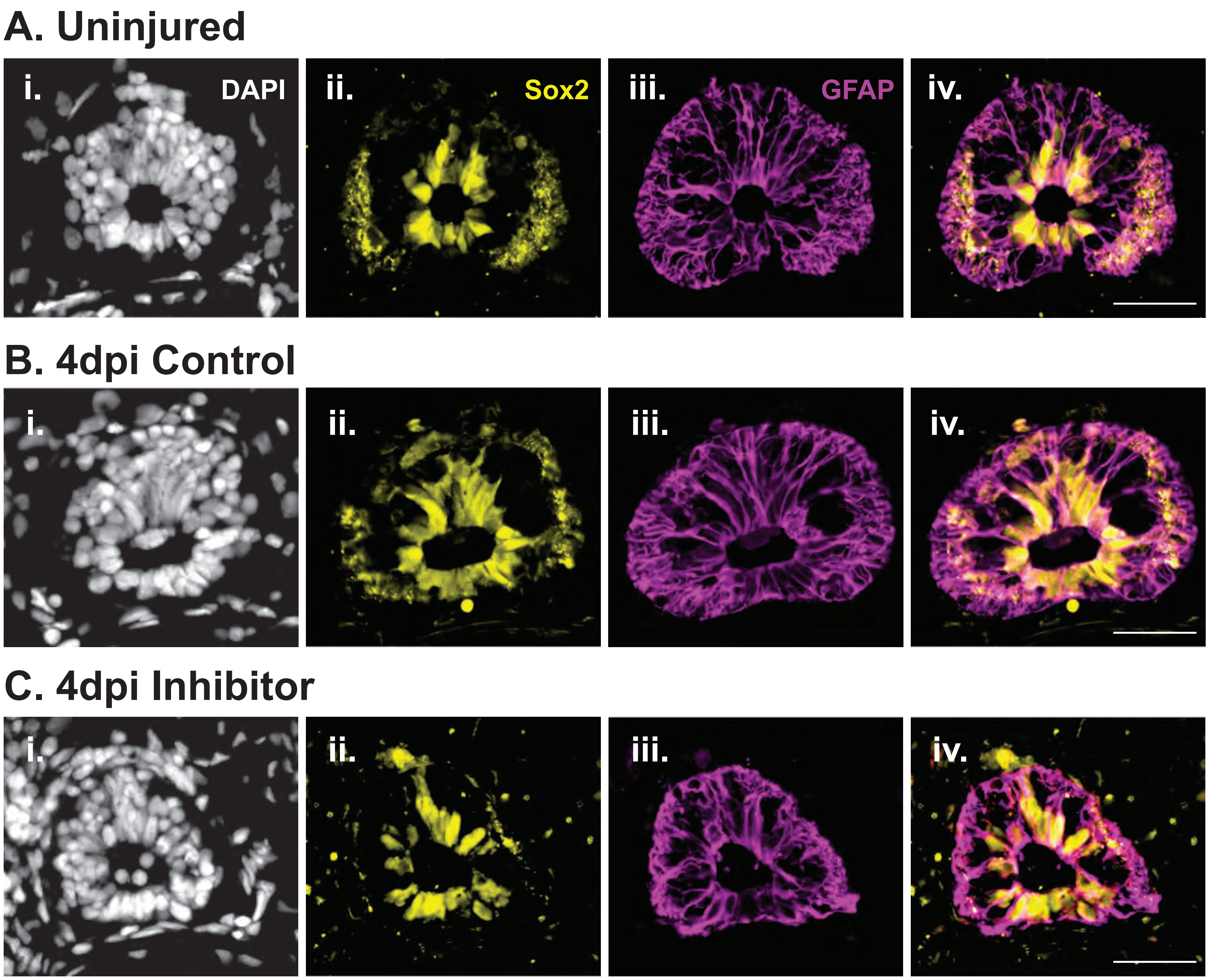

Fig. S2. Cells lining the central canal of the spinal cord co-express Sox2 and GFAP. Immunofluorescence staining of cross-sections of axolotl spinal cord with antibodies against GFAP and Sox2. In (A) uninjured, (B) 4dpi control and (C) miR-200a inhibitor treated samples (n=5), all Sox2+ ependymal glial cells lining the central canal are GFAP+. Scale bar= 50µm.

Development • Supplementary information

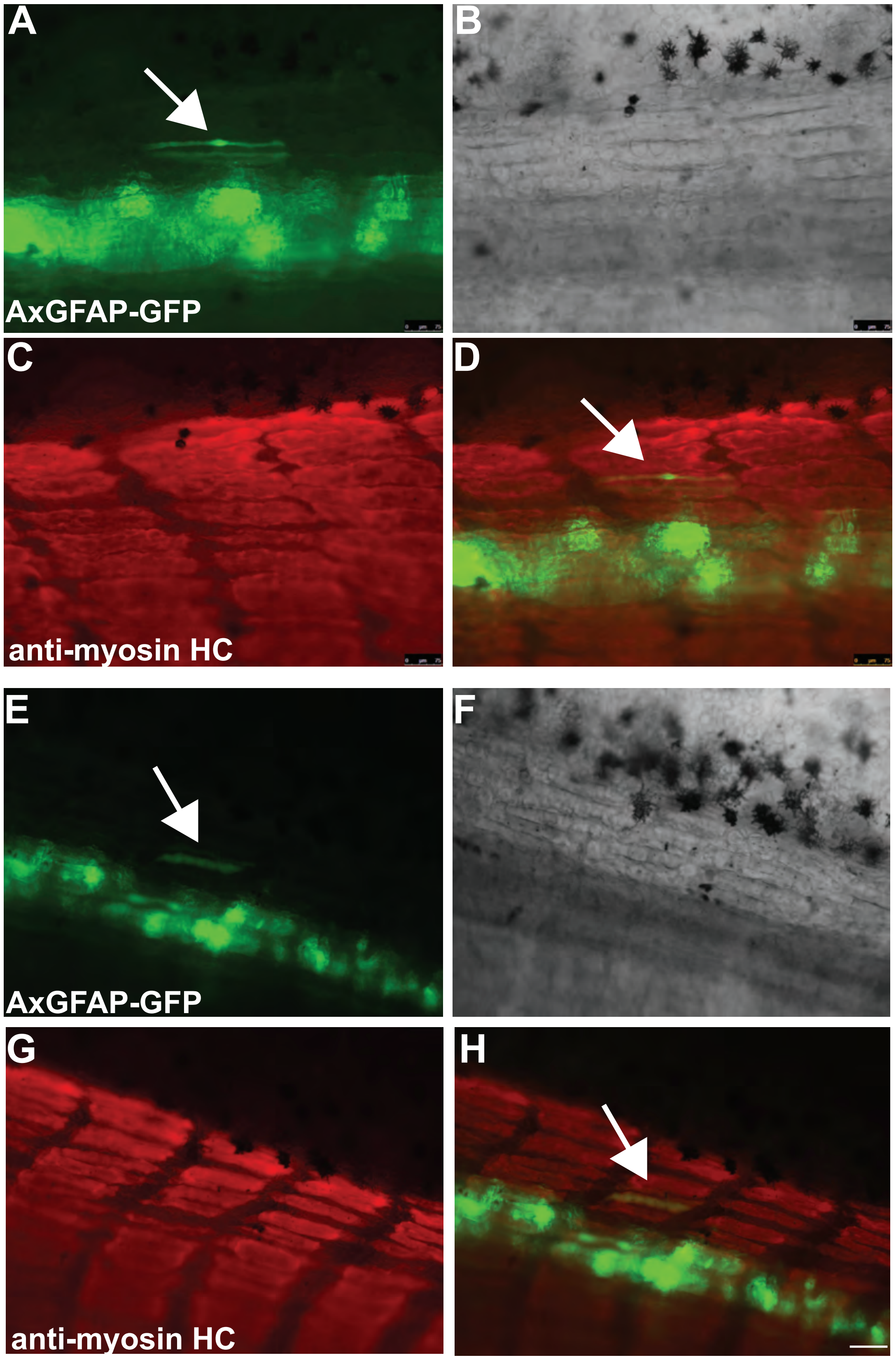

Fig. S3. Cells tracked in miR-200a inhibited animals exit the spinal cord and form muscle. Animals were fixed 14 days post injury. (A-D) Example 1, panel A cells labelled using axolotl GFAP promoter driving expression of GFP, cells within the spinal cord are out of focus, cells which have become muscle but still retain some GFP protein are in focus. (B) DIC image of the animals, (C) anti-myosin HC expression.
(D) Overlay of the GFP with the anti-myosin HC staining. (E-H) Second example of cells exiting the spinal cord and becoming muscle after miR-200a knock-down. Arrows indicate muscle fibers. Scare bar =75µm

Development • Supplementary information

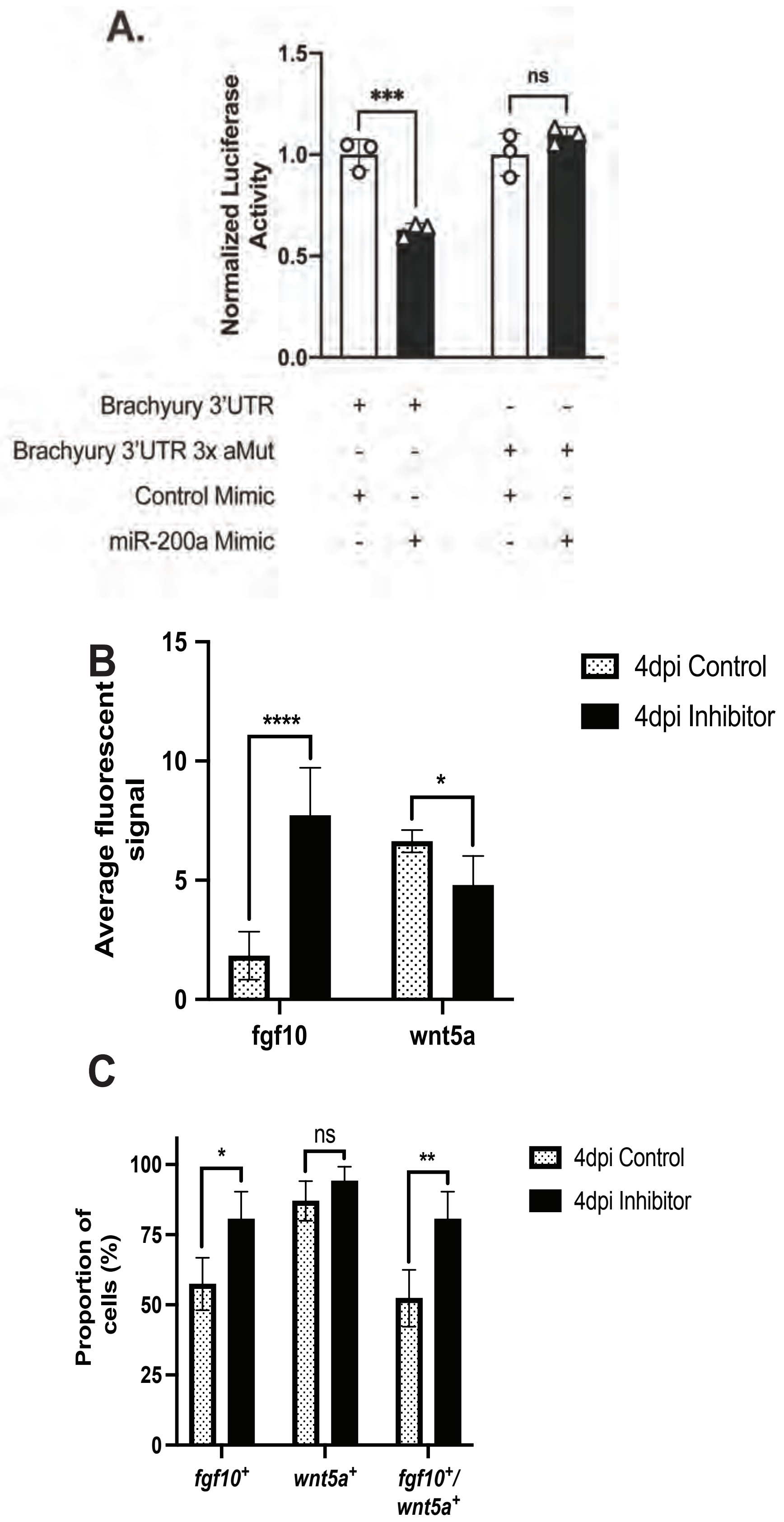

Fig. S4. Multiple miR-200 members directly regulate the brachyury 3' UTR. (A) Co-transfection of B35 cells with a brachyury 3' luciferase reporter and a miR-200a (A) or miR-200b (B) mimic results in decreased luciferase activity compared to the control mimic (n=5). Mutation of all miR-200a seed sequences in the brachyury 3' UTR alleviates this repression suggesting it is a direct target of miR-200 in axolotl (n=4).
(B) miR-200a inhibition dysregulates fgf10 and wnt5a expression in the spinal cord. Quantification of the average fluorescent signal of fgf10 and wnt5a from in situ hybridization experiments (n=6). (C) Quantification of the portion of cells lining the central canal that express fgf10 or wnt5a and of those that express both fgf10 and wnt5 (n=6)
$*p \leq 0.05$, $*** \ p \leq 0.001$, $****p \leq 0.0001$. N.S. is not significant. Error bars represent ±S.T.D.

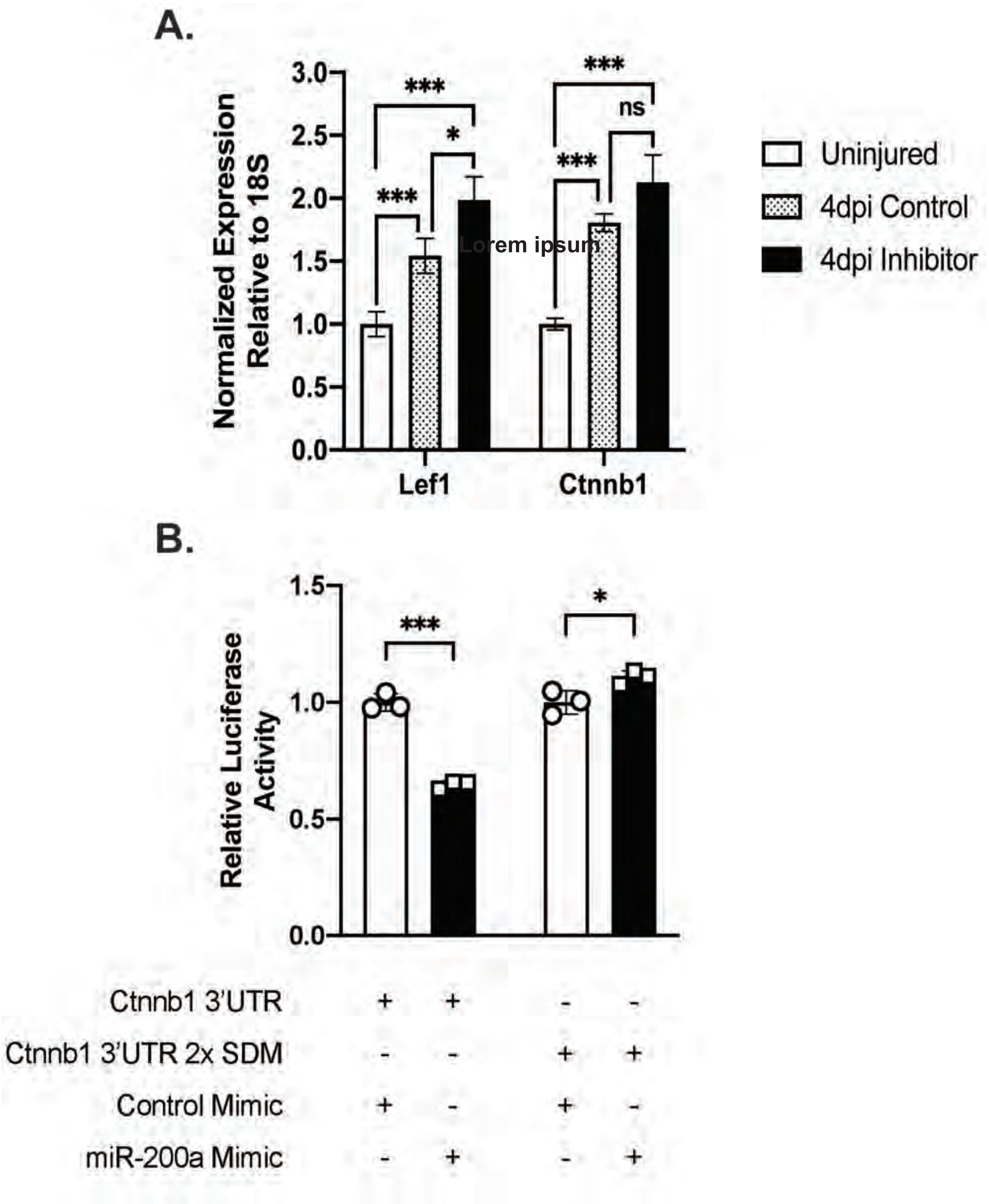

Fig. S5. miR-200a may regulate the expression of Wnt signaling components.
(A) qRT-PCR analysis revealed that miR-200a inhibition leads to increased expression of Wnt signaling transcriptional components lef1 and β-catenin. (B) Co-transfection of B35 cells with a β-catenin 3' UTR luciferase reporter and a miR-200a mimic leads to decreased luciferase activity compared to controls. Mutation of both miR-200a seed sequences in the β-catenin 3' UTR alleviates this repression (n=3). *p≤0.05, *** p≤0.001, N.S. is not significant. Error bars represent ±S.T.D.

Development • Supplementary information

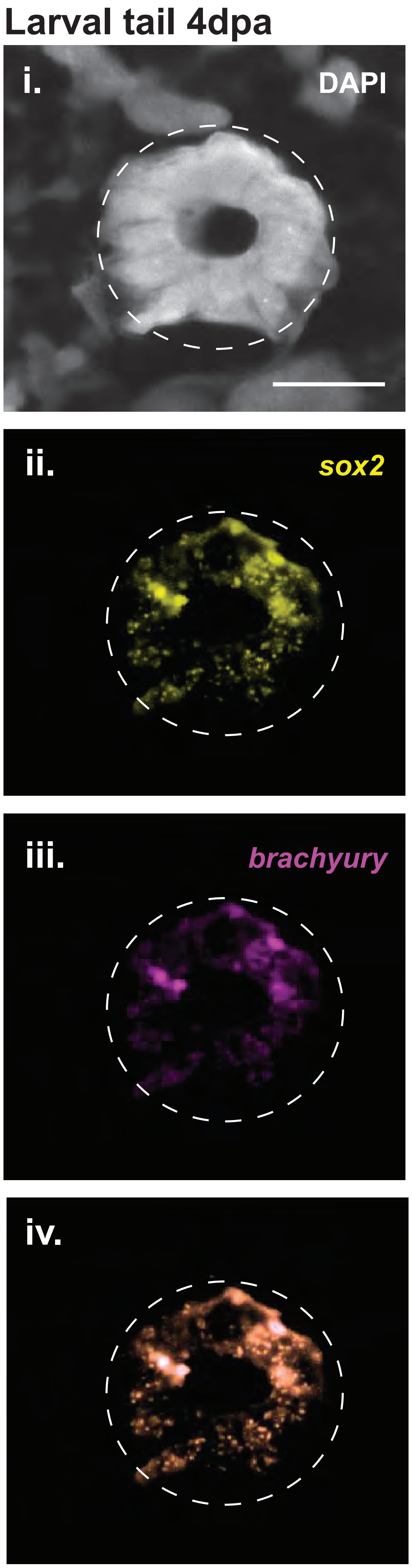

Fig. S6. brachyury is expressed in regenerating spinal cord stem cells following amputation in larval animals. At 4 days post amputation (dpa) both sox2) and brachyury (iii) are expressed in the spinal cord stem cells and share an overlapping expression pattern (iv)(n=6). Scale bar= 50µm.

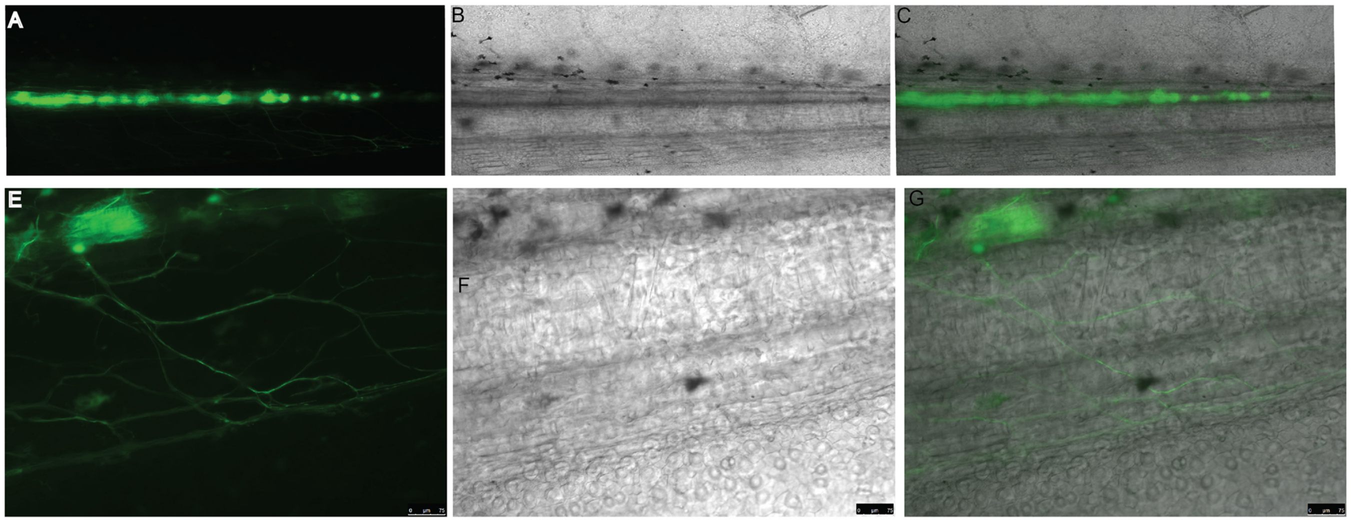

Fig. S7. GFP labelled cells in the spinal cord give rise to new glial cells (A-C), tiled overview image of the regenerating spinal cord 14 days post injury. Calls in the spinal cord can also give rise new neurons during regeneration (E-G) (iv) (n = 6). Scalebar = 50 µm.

Table S1. All TPMs from transcriptional profiling of control and miR-200a inhibitor-treated samples.

Click here to download Table S1
